# Preparation and Characterization of Gelatin-Agarose and Gelatin-Starch Blends Using Alkaline Solvent

**DOI:** 10.3390/ijms24021473

**Published:** 2023-01-12

**Authors:** Oleksandra Dzeikala, Miroslawa Prochon, Anna Marzec, Szymon Szczepanik

**Affiliations:** Institute of Polymer and Dye Technology, Faculty of Chemistry, Lodz University of Technology, Stefanowskiego 16, 90-537 Lodz, Poland

**Keywords:** biocomposites, biopolymers, biodegradation, collagenic proteins, polysaccharides, gelatin, agarose, starch

## Abstract

Plastic waste is a serious problem in modern society. Every day, mankind produces tons of waste that must be disposed of or recycled. The most common types of plastic waste are disposable tableware, bags, packaging, bottles, and containers, and not all are recycled. Therefore, there is a great interest in producing environmentally friendly disposable materials. In this study, modified gelatin blends using polysaccharides (e.g., agarose, starch) were produced to obtain a stable coating. Various techniques were used to characterize the obtained bioplastics, including FTIR spectroscopy (Fourier-transform infrared spectroscopy), TGA (thermogravimetric analysis)/DSC (differential scanning calorimetry), contact angle measurements, and surface energy characterization. We also investigated the influence of thermal and microbiological degradation on the properties of the biocomposite. The addition of agarose increased the hardness of the blend by 27% compared to the control sample without added polysaccharides. Increases were also observed in the surface energy (24%), softening point (15%), and glass transition temperature (14%) compared to the control sample. The addition of starch to the biopolymer increased the softening point by 15% and the glass transition temperature by 6%. After aging, both blends showed an increase in hardness of 26% and a decrease in tensile strength of 60%.

## 1. Introduction

Bioplastics are polymers produced from renewable biomass, such as vegetable fats and oils, corn starch, straw, wood shavings, sawdust, or food waste [1,2]. Bioplastics can be made from agricultural byproducts such as plant leaves or roots as well as products of degradation of used plastic bottles and other containers using microorganisms [3,4,5]. One of the ecological raw materials are of natural origin such as starch, cellulose, or gelatin, with possible modification. Wheat gluten and casein show promise as raw materials for producing biodegradable polymers. Soy protein is another material that can also be used to produce bioplastics. The main difficulty associated with protein products is their water sensitivity [6,7,8,9]. Other problems such as cost and performance restrict the use of biopolymers.

**Figure 1:**
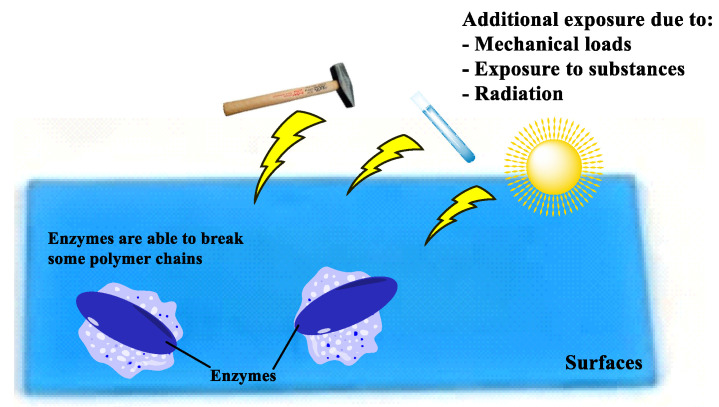
Polymers biodegradation: enzyme attack on plastic surface.

Bioplastics can degrade in a variety of environments, including soil, water, and compost (Figure 1), making them more environmentally friendly than conventional plastics [10,11]. Both the structure and the composition of biopolymers and biocomposites can affect the biodegradation process, so changing the composition and structure can increase biodegradability. Soil and compost are more favorable environmental conditions in terms of biodegradation, due to their high microbiological diversity. Composting not only effectively breaks down bioplastics, but also significantly reduces greenhouse gas emissions [12]. The biodegradability of bioplastics in composting environments can be increased by adding soluble polysaccharides [13]. The soil environment contains a large variety of microorganisms, which contributes to the biodegradation of bioplastics. Unmodified materials require higher temperatures and longer biodegradation time. Some bioplastics break down more efficiently in water. However, this poses a threat to aquatic ecosystems [10,11].

Gelatin is a compound formed by collagen denaturation. The composition of gelatin differs from that of undenatured collagen in terms of its amino acid composition. Gelatin consists of completely denatured protein with Mw = 50–150 kDa [14,15]. Chemical denaturation is performed by hydrolysis catalyzed by enzymes, acids, or bases. Thermal denaturation takes place under mild conditions by heating the collagen in neutral or slightly acidic conditions to about 40 °C. The transition is clear and occurs in a few minutes within a small temperature range. Only the hydrogen bonds and hydrophobic bonds are broken, which helps to stabilize the collagen helix, causing the dissociation of collagen fibers into tropocollagen molecules. The intramolecular bonds between the three helix chains are broken, resulting in a mixture of proteins and amino acids called gelatin [16,17].

Polysaccharides are another example of biopolymers. Sourced from plant matter, they can possess different structures depending on method of their acquisition, species of the plant, and different modifications. Though there is a huge variety in chemical structure of polysaccharides, the main block of the polymeric chain is glucose [18,19]. The hydroxyl and carboxyl groups of the glucose can easily create hydrogen and ion bonds with other particles [20].

Survyati et al. [21] describe the preparation of a gelatin biopolymer with the addition of chitosan and glycerol. The blend had high water absorption and a high degree of swelling. Hydroxyl and carbonyl functional groups indicate high biodegradability and environmentally friendly properties. Mroczkowska et al. [22,23] describe environmentally friendly packaging made from a mixture of gelatin and starch. The renewable bioplastics were characterized by relatively short degradation times under moderate conditions (20 °C) in soil, with no adverse effect on living organisms.

To modify physicochemical properties of the gelatin blends, the addition of polysaccharides such as agarose and starch have a great potential as their developed helix structure will improve the matrix of the blend. Modification of gelatin by adding starch and agarose to produce mechanically durable films will be possible by changing the pH of the environment to alkaline, in which the formation of intermolecular interactions through the formation of stable hydrogen bonds is much more favorable for polysaccharides. This can be ensured by the appropriate concentration of sodium hydroxide used in the synthesis process using the solvent casting method. In the literature, the increase in physicochemical properties, including, for example, the melting point, is due to the introduction of polysaccharide derivatives (sucrose and transglutaminase); therefore, agarose and starch were used in the work due to their better availability [24]. The polysaccharides used in the research will strengthen the gelatin film due to their developed structure, the availability of functional groups, and the increase in the melting and softening points of the prepared systems. The melting point of gelatins, agarose, and starch are 27–32 °C, 85 °C, and 70–84 °C, respectively. The solidification point for gelatin, agarose, and potato starch is 15 °C, 32–42 °C, and 53 °C, respectively. Therefore, the addition of polysaccharides should result in increased thermal and mechanical stability of gelatin composites [25,26].

The aim of this research was to create ecologically friendly gel material based on proteins of collagen origin with polysaccharide additives. Such gelatin–starch and gelatin–agarose blends have not been described in the literature for disposable one-use products so far. Moreover, as opposed to plastics, gelatin blends use renewable sources and biopolymeric raw materials that give them desired biodegradable properties and do not require dangerous solvents and substrates for their production.

## 2. Results and Discussion

### 2.1. FTIR Spectroscopy

**Figure 2:**
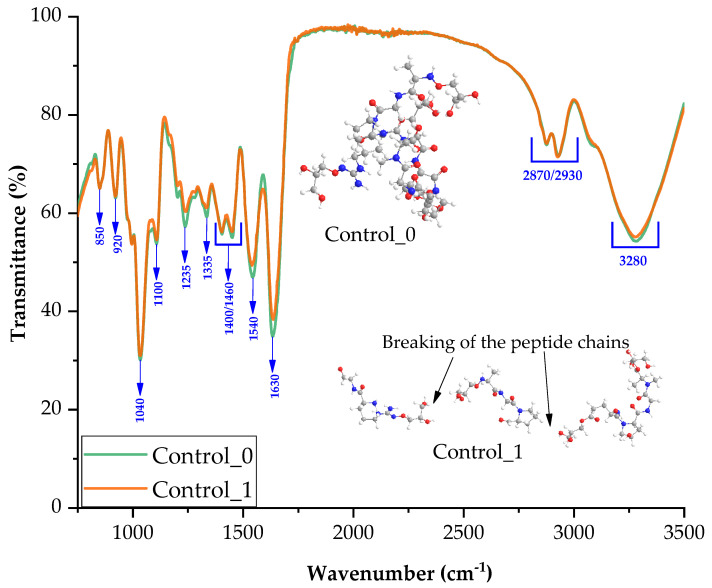
FTIR spectra of samples: Control_0 and Control_1.

Figure 2 shows the graphical spectrum from FTIR spectroscopic (Fourier-transform infrared spectroscopy) analysis for Control_0 and Control_1. To determine structural changes, we compared the spectra for the control composition containing only gelatin and glycerin as well as the synthesis in varying concentrations of alkaline medium.

Introducing a base during synthesis of the blend did not significantly change the structure of blends. Most of the peaks in both spectra are from gelatin. According to the literature, interactions derived from functional groups at wavenumbers 1630, 1540, 1460 cm^−1^ are derived from gelatin, and correspond to carbonyl, amide, and methylene bonds [27].

Groups derived from glycerin were observed on the FTIR spectra: stretching -OH at 3280 cm^−1^ and bending -OH at 920 cm^−1^ [28,29]. The -C-H stretch bond was revealed by peaks in the region 2870–2930 cm^−1^ [30]. A signal from the -COH moieties is seen in the range of 1400–1420 cm^−1^ and the signal from the -C-O- stretching moiety of the primary alcohol is visible at 1100 cm^−1^ [31,32].

Figure 2 shows the possible reaction mechanism between gelatin and glycerin. Hydrogen derived from glycerin enables the formation of hydrogen bonds inside the blend. The incorporation of the base into the composition resulted in the breaking of the peptide chains.

**Figure 3:**
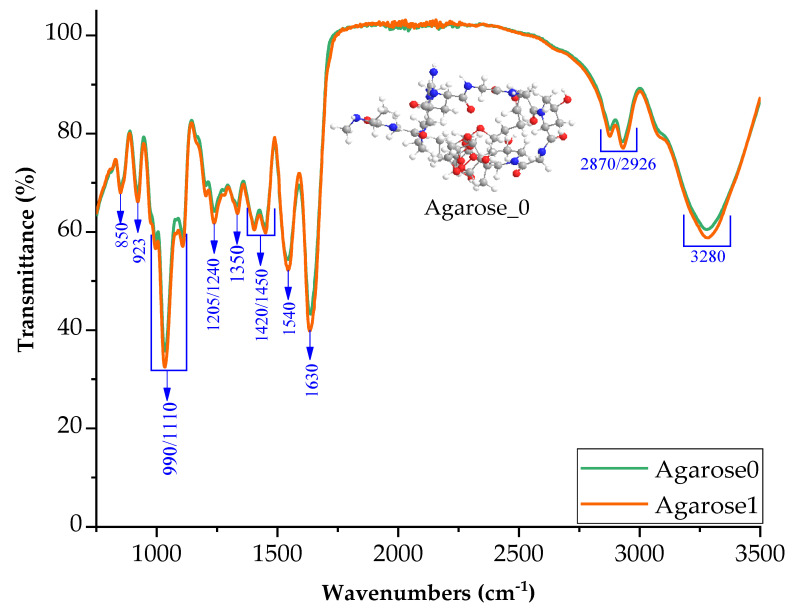
FTIR spectra of samples with agarose additive in neutral and basic pH (1M NaOH).

The obtained spectrum shows that the introduction of agarose did not affect the appearance of new interactions related to the appearance of new absorption bands (Figure 3). This is probably because agarose has the same functional groups as gelatin and glycerin [33].

The introduction of the active base into agarose could cause breakage of the bond between the hydrogen and oxygen, therefore creating electronegative oxygen that potentially partakes in reactions and generates ionic bonds.

**Figure 4:**
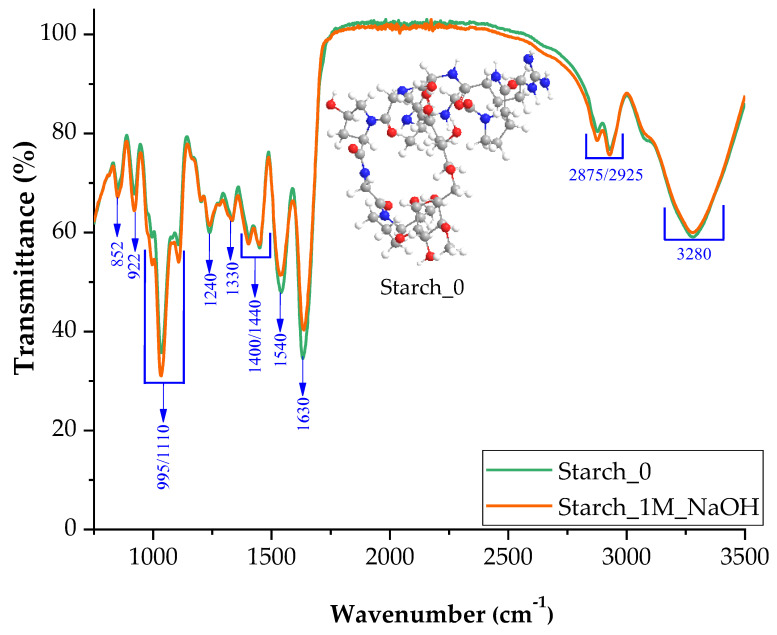
FTIR spectra of samples with starch additive in neutral and basic pH (1M NaOH).

Figure 4 shows the FTIR spectrum for the blend with the addition of starch. As in the case of agarose, no significant changes in interactions in the polymer were observed. Figure 4 shows the possible interactions between starch and the gelatin blend.

The introduction of a base during synthesis of the blend probably influenced the structure of the starch as well as the structure of agarose, causing the formation of an oxygen radical.

**Figure 5:**
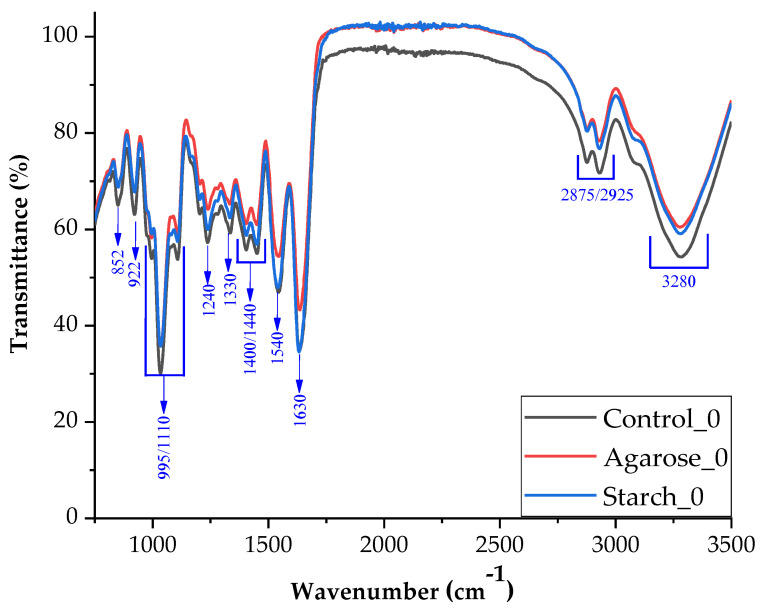
FTIR spectra of samples control and with additives (starch, agarose) in neutral pH.

Figure 5 shows the comparative spectra for the control sample and samples containing the addition of polysaccharides in their structure without modification with sodium hydroxide. Figure 5 shows that all three spectra have the same peaks but differ in their intensity.

The amide A band resulting from N-H stretching and free O-H was observed at a wavelength of 3270 cm^−1^, the C-H stretching bond for amide B at 2925 cm^−1^, the C=O bond stretching with vibration C-N bonds was from amide I at 1630 cm^−1^ and bending NH with C-N stretching vibrations for amide II at 1540 cm^−1^. Amide III represents the vibration in the plane of the C-N and N-H groups of the bound amide or the vibration of the CH_2_ group (1400–1540 cm^−1^). The appearance of the peak derived from amide I indicates that the composites made mainly assume the α-helical configuration, which confirms the appearance of amide II at 1540 cm^−1^. The band at a wavelength of 1330 cm^−1^ corresponds to the vibration originating from the proline side chains. O-H bending bonds derived from glycerin were observed at 922 cm^−1^ [34,35].

### 2.2. Mechanical Properties

Table 1 shows the mechanical properties of hardness, tensile strength, and elongation at break of the samples. It also shows the hardness of the base blend (without polysaccharidal additives) and the blends with additives. The hardness of samples was determined on the Shore’s “A” scale. A Zwick Roell testing machine was used to determine the tensile strength (T_S_, MPa) and relative elongation at break (E_b_, %) 3. All the results are showed alongside with their standard deviations.

As can be seen in Table 1, the hardness of the blend decreased with increasing base concentrations during synthesis. These changes may have been caused by the fact that with increasing sodium hydroxide concentrations, the connections in collagen compounds derived from gelatin are probably broken (Figure 1). Proline and hydroxyproline amino acids may be cleaved from the main polymer chain, having a major impact on the hardness of the obtained blends.

Comparing the results for the polysaccharide samples to the base sample, the hardness first increases to its maximum at a NaOH concentration of 0.5M, and then decreases at higher concentrations. These changes can be explained by the fact that the sodium hydroxide introduced into the blend during the synthesis reacts for the most part with the polysaccharide, causing the formation of covalent bonds. Due to the creation of those bonds and reactions, the polymer chain is extended, causing it to branch out as well. It is also possible for branching to become entangled [36]. Increasing concentration of sodium hydroxide to 1M, the hardness of the samples decreases due to the higher concentration of the base and the covalent bonds are broken with alkaline solvent, therefore creating a softer matrix because of its lower presence of cross-linkage.

Maiko Iwaki et al. [37] summarize the results of research on the effect of methyl mercaptan on silicone-added dentures. The average value for MMP samples is in the range of 19.6–23.6 degrees. The research conducted by Prinya Chindaprasirt and Ubolluk Rattanasak [38] described the synthesis of alkali-activated porous fly ash composites (AA-fly ash). The Shore A hardness scores for these composites are 35 degrees without aluminum and 90 degrees with 1.5 parts aluminum. The publication "Antifungal activity and Shore A hardness of a tissue conditioner incorporated with terpinen-4-ol and cinnamaldehyde" [39] determined the hardness of a tissue conditioner (Softone™) modified by the inclusion of terpinen-4-ol and cinnamaldehyde. For the control sample (non-modified Softone tissue conditioner), the hardness value is 4.96+-0.69 degrees. Juan Carlos Garcia-Quesada and others in the article entitled "Processability of PVC Plastisols containing a polyhydroxybutyrate-polyhydroxyvalerate copolymer" [40] presented the results for Plastisols based on polyvinyl chloride (PVC) with the addition of PHA. The hardness results for the PVC blend with polyhydroxybutyrate and polyhydroxyvalerate copolymer range from 70 to 95 degrees. The publication "Resins-based denture soft lining materials modified by chlorhexidine salt incorporation: An in vitro analysis of antifungal activity, drug release and hardness" [41] describes the change in Shore’s scale A hardness of soft denture lining materials based on resins derived from poly(methyl methacrylate) (PMMA) or poly(ethyl methacrylate) (PEMA) containing chlorhexidine diacetate (CDA) or chlorhexidine hydrochloride (CHC). For PMMA, the Shore hardness is about 10 degrees, while PEMA is in the range of 7–13 degrees.

As the concentration of base (NaOH) increased, the tensile strength decreased, and the elongation at break increased. This behavior can be explained by the fact that as the concentration of the base increases, the bonds inside the collagen chain are broken. Branched fragments of macromolecules probably become entangled and new nodes are formed. When stretched on a testing machine, the tangled fragments stretch out and become disentangled. The force needed to tear the sample is therefore much lower than in the case of the base sample (without the addition of a base of a given concentration) and its relative elongation is higher.

The results show that with the increasing concentrations of the base in the blends containing polysaccharides, the tensile strength increased, reaching the highest value at a concentration of 0.5M of base. The tensile strength then decreases with 1M NaOH. A similar increase in the elongation at break was observed up to 0.5M, followed by a decrease.

The blends with the best mechanical properties were selected for further analysis. These were the compositions with the addition of 0.5M base, Control_0.5, Agarose_0.5 and Starch_1.

### 2.3. Contact Angle and Surface Free Energy

Surface free energy (SFE) was determined using the OWRK method. The contact angles of three liquids (water, diiodomethane, and glycol) were measured on the surface of samples synthesized in the selected alkaline medium (NaOH 0.5M). The surface layer indicates the morphology of the layer itself and its electrical properties. The results were used to determine the values for surface energy. The data are presented in Table 2.

Contact angle tests are based on surface tension forces. Atoms at the interface are subject to a different system of forces than the atoms at the end of the phase. The atoms at the interface are attracted by the atoms at the back of the phase and by the atoms from the adjacent phase, so they are in an asymmetric system of forces.

The covalent interatomic bonds in the studied blends may be polarized to varying degrees. The degree of polarization depends on the difference in the electronegativity of the atoms making up the bond.

Water is a liquid with high surface tension. It has a relatively large contact angle if it is on a substrate with low surface energy. This was the case with the sample containing agarose. The addition of starch resulted in a decrease in surface tension. This, in turn, facilitated the spread of the droplets and caused a reduction in the contact angle.

As can be seen in Table 2, Agarose_0.5 was characterized with having the highest SFE. This is probably related to the formation of macromolecular connections due to covalent interactions resulting from the interactions of the -OH moiety derived from agarose.

The blends were characterized by high polarity and e macromolecules with atoms capable of forming hydrogen bonds. There were strongly polar carbon–nitrogen interactions, as well as strongly polar -NH- and -(C=O)- groups. There were also weak intermolecular interactions between these groups and hydrogen bonds.

The interaction between the surface of the material and the hydrophobicizing agent affected the degree of adhesion. Table 2 shows that the adhesive forces of the samples were very low and may not prevent water absorption.

The surface roughness of the samples also affected the contact angle. The blends were characterized by more pits and bubbles. The blend containing agarose had the highest contact angle with the polar liquid.

The roughness of the samples increased the number of polar groups with a larger contact angle in the case of water than in the case of diiodomethane [42].

### 2.4. Thermal Analysis

Differential scanning calorimetry (DSC) and thermogravimetric analysis (TGA) were performed for selected samples to determine the magnitude of thermal transformations and the temperature ranges at which they occurred. Phase transformations like melting point, glass transition etc., were examined by means of TGA.

**Figure 6:**
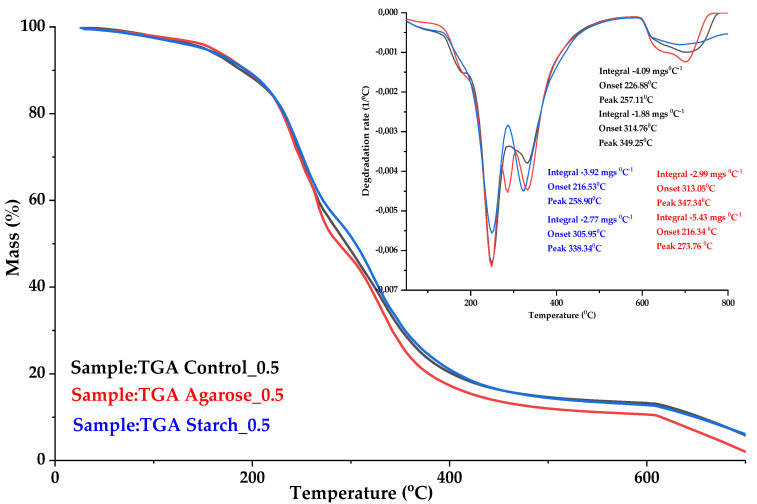
Thermogravimetric analysis curves (TGA) of gelatin samples: Control_0.5 (black curve); Agarose_0.5 (red curve); Starch_0.5 (blue curve).

**Figure 7:**
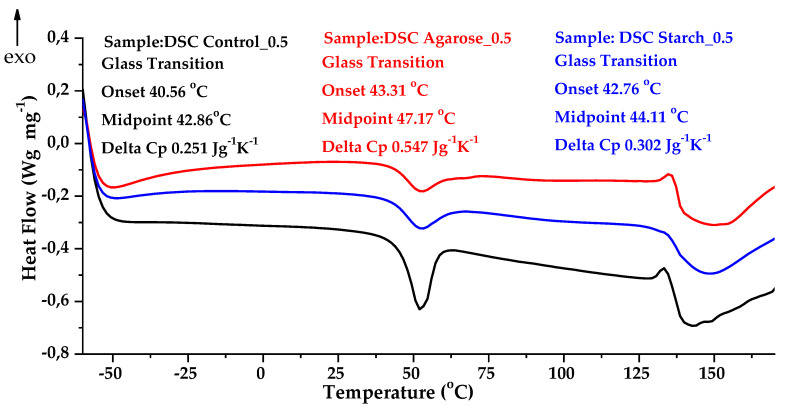
Comparison of DSC analysis curves for gelatin samples: Control_0.5 (black curve); Agarose_0.5 (red curve); Starch_0.5 (blue curve).

Figure 6 and Figure 7 show the thermograms for TGA and DSC, respectively, for the control sample and samples with the addition of polysaccharides.

Differential scanning calorimetry showed that the glass transition temperature T_g_ for the Control_0 sample started at 46.25 °C. For the sample supplemented with agarose with an alternate 0.5M base, T_g_ started at 47.17 °C and the caloric capacity ∆C_p_ was 0.547 (J/g∙K). The increase in temperature was probably related to the increase in cross-linking due to the formation of new connections, which limited changes of the positions of mers and segments of polymer chain in the macromolecules.

To determine the softening point, a Vicat analysis was performed for various biopolymer blends. The results are presented in Table 3. The softening point increased by 5 °C in the case of blends with the addition of polysaccharides. It is likely that the improvement in heat resistance was associated with an increase in the crystalline phase and an increase in T_g_.

The addition of agarose to the gelatin composition raised the heat capacity to 0.547 (J/g∙K). In comparison, the heat capacity of the control sample was 0.251 (J/g∙K). This may indicate an increase in cross-linking due to the formation of new connections which limited changes of the positions of mers and segments of polymer chain in the macromolecules.

### 2.5. Scanning Electron Microscopy (SEM)

**Figure 8:**
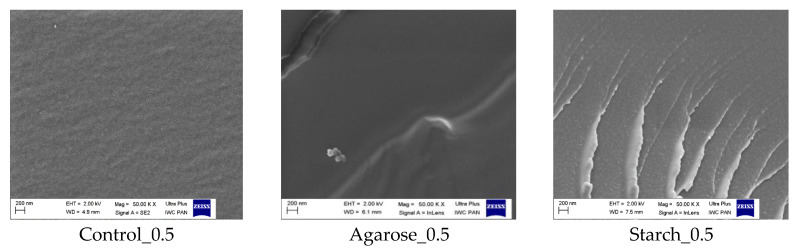
Scanning electron microscope images of gelatin blends.

The morphology of the selected samples was assessed based on SEM images of the breakthroughs of individual materials (Figure 8). Photographs at 50,000× magnification enabled the diverse morphology of the analyzed materials to be observed. The Control_0.5 blend without additives was used as a reference sample for the blends with the addition of polysaccharides: Agorose_0.5 and Starch_0.5.

The SEM images show that the control sample is homogeneous and structured. Fine spherical structures are visible on the surface. These may be the ends of collagen filaments, formed into clusters resulting from the reorganization of the heliacal structures that make up the polypeptide chains of scleroproteins. In contrast, a small number of fine agglomerates, possibly consisting of undissolved agarose, were observed in the blend with the addition of agarose. The images of the samples with the addition of starch show structures resembling scratches, which may be starch deposits on the surface of the blend.

### 2.6. Equilibrium Swelling Analysis

To determine the degree of cross-linking in the blends, equilibrium swelling was measured. The samples were placed in water for 48 h. After removal from the solvent, it was noticed that the samples had increased their volume several times.

Table 4 shows the calculated equilibrium weight swell Q_w_ and the degree of cross-linking α_c_ for the samples that did not dissolve during the measurement. The control blend with the addition of 0.5M NaOH (Control_0.5) was characterized by the lowest equilibrium swelling value. This was likely due to solvent molecules penetrating the matrix more easily, thus breaking some bonds. The blends with the addition of polysaccharides showed a much lower hygroscopic effect, and at the same time, a higher degree of cross-linking. These samples had a stable network structure. The introduction of additives had a positive effect on the degree of cross-linking of the samples. As the degree of cross-linking increased, the flexibility decreased and the resistance to permanent deformation and creep increased.

### 2.7. Biodegradability and Thermo-Oxidative Aging of Samples

#### 2.7.1. Thermo-Oxidative Aging

**Figure 9:**
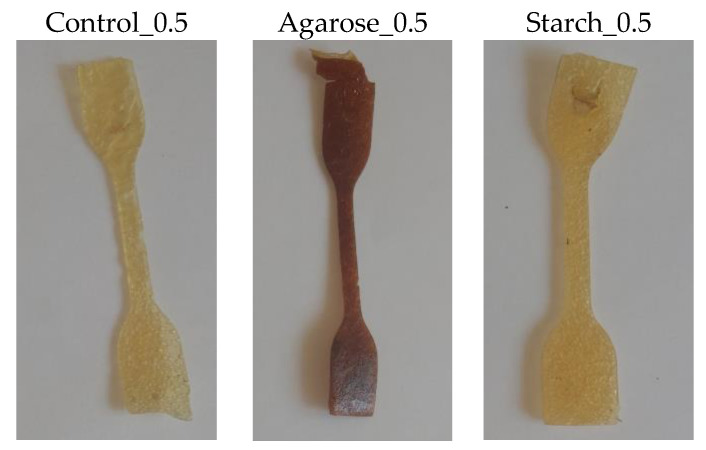
Samples after thermo-oxidative aging.

To determine the resistance of the samples to thermo-oxidative aging, accelerated aging measurements were performed. The samples were placed in a thermo-oxidation chamber for 7 days. Figure 9 shows the samples following the aging process.

Based on the appearance of the samples after the thermo-oxidative aging process, organoleptically aging did not significantly affect the surface appearance of the samples. The samples showed slightly decreased flexibility.

#### 2.7.2. Determination of Color Change after Aging

The color change was measured in the CIELAB space using a UV–VIS spectrophotometer. The samples before aging were used as standard samples. The results of the color change parameter dE∗ab and color changes on the surface of samples are presented in Table 5. The dE∗ab parameter describes the differences in color compared with the samples before aging.

The blend containing starch showed the lowest color change. The highest color changes were observed in the blend with the addition of agarose. This was probably related to the difference in the structure of agarose and starch. In the structure of agarose, there are CH_2_OH groups on the galactose ring [43].

#### 2.7.3. Determination of Changes in Mechanical Properties after Aging Processes

Table 6 presents the Shore hardness scores H, tensile strength T_S_ (MPa), and the relative elongation at break E_b_ (%) for the blends following thermo-oxidative aging.

Table 6 presents the results of the analysis of mechanical properties after thermo-oxidative aging. The tensile strength (Ts) of all samples increased after the aging process, but the relative elongation at break (E_b_) and hardness (H) decreased. This may be due to the formation of oxygen radicals, which lead to secondary cross-linking of the matrix during the thermo-oxidative aging. The formation of the secondary network significantly affects the mechanical properties of the materials.

#### 2.7.4. Determination of the Change in Contact Angle after Aging Processes

**Figure 10:**
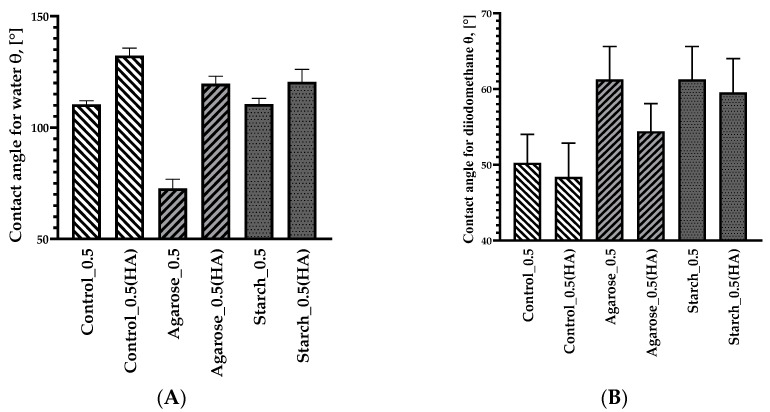
Contact angles of water (**A**) and diiodomethane (**B**) on the surfaces of selected blends (0.5M NaOH) after thermo-oxidative aging.

A goniometer was used to determine the surface energy of the aged samples. The results are shown in Figure 10 for the polar liquid (A) and the non-polar liquid (B).

Figure 10 shows the results of the contact angles for samples subjected to thermo-oxidative aging for 7 days. The contact angles for the polar liquid (distilled water) increased for all mixtures after aging, ranging from 114° to 135°. This indicates a change in the structure and formation of more hydrogen bonds due to the presence of free radicals in the matrix. The greatest change in the contact angle was observed for the sample with the addition of agarose reaching 123.15°. On the other hand, for the non-polar liquid (dioiodomethane), there was a decrease in contact angles for all blends, which may indicate a decrease in non-polar groups (amine, cyano, etc.)

### 2.8. Microbial Aging

To determine the resistance of the samples to microbiological aging, a test was carried out in accordance with the PN-EN ISO 846 standard. Universal soil, which has a pH of 6.0–7.0, was used. Three paddle-shaped samples of each type of blend were placed on a layer of soil in a container and then covered with another layer of soil. The containers were placed in a climatic chamber for 30 days at 30 °C with humidity of 80%.

**Figure 11:**
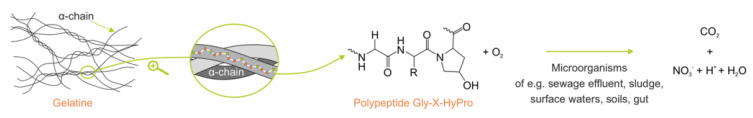
Degradation of gelatin blends and products due to microbial aging visualized by Melanie Baumgartner et al. in their work “Resilient yet entirely degradable gelatin-based biogels for soft robots and electronics” published in Nature Materials, 19(10), page 1102–1109 [44].

After the completion of the measurement, complete biodegradation of the blends was observed. This is consistent with a previous study in the literature [44], in which biodegradation leading to complete demineralization of created gelatin blends was observed within 10 days. Figure 11 shows the distribution of gelatin blends under the influence of soil microorganisms.

#### Mass Change of Samples after Biodegradation

**Figure 12:**
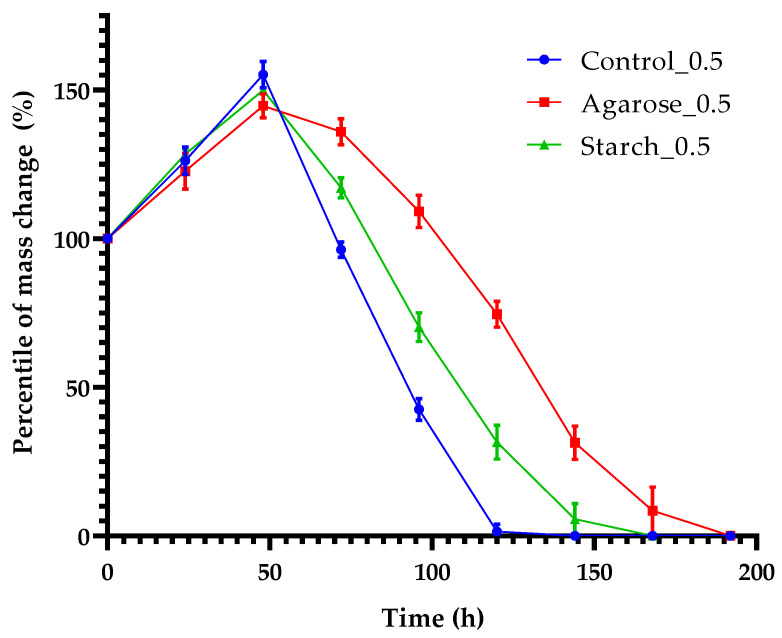
Percentile of mass change in time.

To determine the effect of microorganisms on degradation of blends, the analysis of mass change of the samples was carried out to determine the microbiological aging of gelatin blends. The results are shown in Figure 12.

Figure 12 shows that, in 24 h, all samples increased in weight, which is related to the gelatin’s ability to absorb water from the environment. After the increase in weight, a decline in mass due to degradation of samples by microorganisms can be observed. The decline of control sample’s mass was faster than other samples. Complete decomposition occurred within 144 h and a similar result was obtained for the sample with starch additive. The sample containing agarose decomposed for the longest amount of time (192 h). Such rapid decomposition of the blends may indicate that they provide a nutritional source for soil microorganisms.

## 3. Materials and Methods

### 3.1. Materials

The following raw materials were used to prepare the polymer matrix: gelatin with an index of 200° Bloom units (pH 5–8, Mw 30 kDa, FoodCare Sp. Z.o.o., Zabierzów, Poland); glycerin (d = 1.26 g/cm^3^; Mw 92 kDa; pH 6–8; flash point 160 °C, CHEMPUR, Piekary Śląskie, Poland); sodium hydroxide (pH: approx. 13–14; solidification point 12 °C, Mw 22 mPas at 40 °C, EUROCHEM BGD Sp. Z o.o. Tarnów, Poland).

The following polysaccharides were added to improve the performance properties of the gelatin blends: agarose (solidification point 80–95 °C, solubility in water ~10 g/L at 80 °C, TAR GROCH FIL Sp. J., Filipowice, Poland) and potato starch (Mw 162 kDa; water solubility 50 g/dm^3^, bulk density 280 kg/m^3^ Kupiec Sp.z.o.o., Krzymów, Poland).

### 3.2. Preparation of the Blends

#### 3.2.1. Preparation of the Polymer Matrix

**Figure 13:**
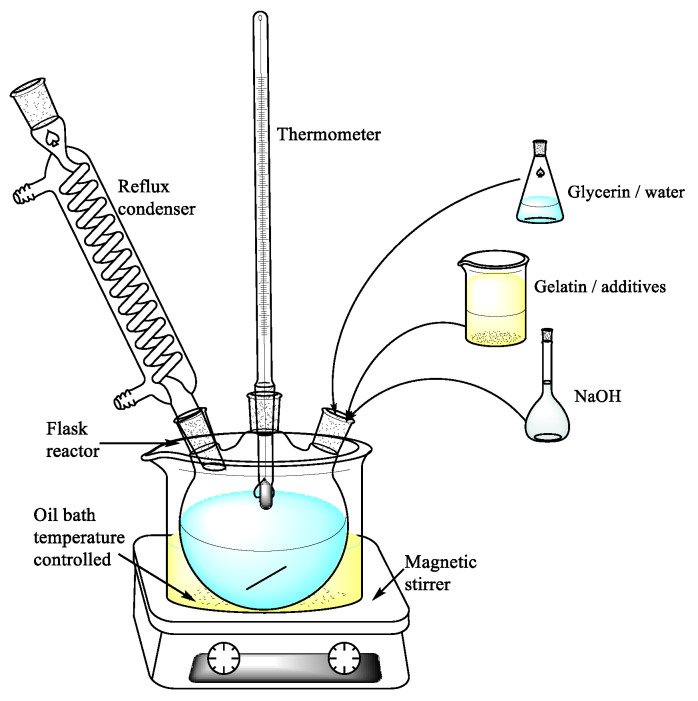
Method of blend synthesis.

The base composition was synthesized in a reaction set consisting of a three-necked flask, a thermometer, a magnetic stirrer, and a reflux condenser (Figure 13). The reaction was performed at 75 °C in an oil bath and was completed when a homogeneous mass was obtained. Components used for blends’ synthesis are presented in Table 7 and Table 8.

Once all the ingredients were dissolved in the three-necked flask, the reaction mixture was thermostabilized in a thermal chamber for 48 h at 85 °C (Binder GmbH, Tuttlingen, Germany). To obtain a homogeneous shape and thickness, the samples were formed using a Skamet 54436, SKAMET, (Skarzysko-Kamienna, Poland) hydraulic press then cut into addles measuring 70 mm in length, 4–5 mm in thickness and 210 mm in width.

Gelatin blends were made by adding glycerin as a plasticizer and sodium hydroxide at the following concentrations: 0.1M (Control_0.1); 0.5M (Control _0.5); 1M (Control _1). The compositions of the synthesized samples are presented in Table 7.

#### 3.2.2. Polysaccharides as Additives

**Figure 14:**
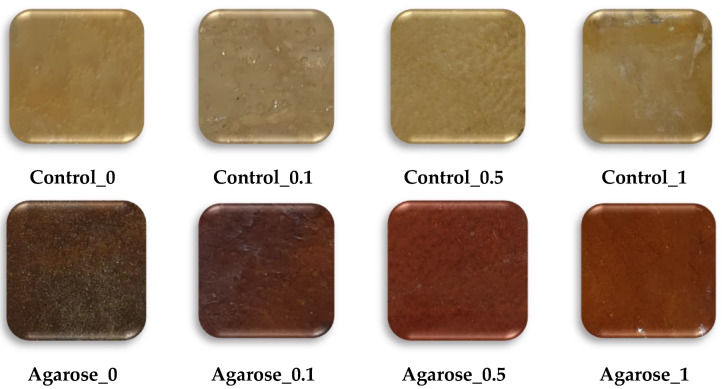

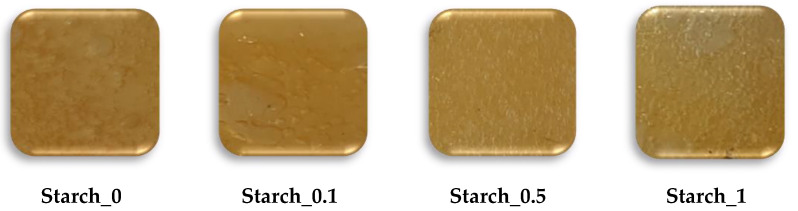
Synthesized blends (CanoScan 4400F).

The base blends (Control_0, Control_0.1M, Control_0.5, and Control_1) were supplemented with polysaccharides (agarose and potato starch) to modify the physicochemical properties of the polymer matrix by weight. The incorporation of 5 parts by weight of the additive was sufficient to determine changes in the base blends. The blend preparation process was the same as that described in Section 3.1. The ingredients are listed in Table 2, where the amounts are presented in parts by weight. Figure 14 shows pictures of the obtained blends made with a CanonScan 4400F camera (Canon Inc., Tokyo, Japan).

### 3.3. Methods

#### 3.3.1. Fourier Transform Infrared Spectroscopy

A Nicolet 6700 Fourier transform infrared (FTIR) spectrophotometer (Thermo Scientific, Waltham, MA, USA) was used to characterize the blends and their structures. Measurements were carried out in the radiation wavelength range of 4000–400 cm^−1^ with a resolution of 0.25 cm^−1^.

#### 3.3.2. Surface Morphology

Surface morphology studies were performed using a Zeiss Ultra Plus Bruker scanning electron microscope (SEM) (Billerica, MA, USA) at 10 kV and magnification was at 50,000× [45,46]. The test samples were prepared as follows: samples were fractured in liquid nitrogen and sputtered with carbon.

#### 3.3.3. Mechanical Properties

Mechanical properties were determined using a Zwick 1435 testing machine (Zwick/Roell, Radeberg, Germany) and a Shore. To determine the static mechanical properties, test samples were cut out from extruded trips with a thickness of 1.6–1.8 mm and length of 150 mm. The measurement conditions were a preload of 0.1 N and a test speed of 50 mm/min. The parameters tensile strength (MPa) and elongation at break (%) (E_b_) were determined. Shore’s scale method was used to determine the hardness of the materials. This method is one of the empirical tests. It measures the initial depth and the depth of the indentation after a certain amount of time. A digital Shore type A hardness tester was used for the analysis. A hardness tester with a pressure force of 12.5 N and a 35 °Sh indenter (Zwick/Roell, Herefordshire, UK). A sample with a thickness of about 4 mm was placed on the hardness tester stand, and then the indenter of the apparatus was lowered onto the surface of the sample. The measurement was repeated in five different places on the surface of the material. The result with relative error was calculated from the average of all measurements. Hardness in this method is presented in Shore’s degrees ºSh.

#### 3.3.4. Thermal Properties

Differential Scanning Calorimetry (DSC) was used to estimate the glass transition temperature (T_g_) and determine changes in the heat capacity (∆C_p_; J/g; K) of selected blends. Measurements were performed with a Mettler Toledo TGA (thermogravimetric analysis)/DSC (differential scanning calorimetry) computer program (Mettlet Toledo, Greifensee, Switzerland) calibrated with a standard (indium or zinc). The samples (4–10 mg, placed in 100 µL-volume open aluminium crucibles) were heated from 0 to 200 °C at a rate of 10 °C/min under an argon atmosphere. Then, the gas was switched from argon to air (flow rate 50 mL/min).

#### 3.3.5. Surface Free Energy and The Degree of Cross-Linking

Surface free energy (SFE) was determined by the OWRK method (Owens Wendt Rabel Kealble) using an OCA 15EC goniometer (Dataphysics, Filderstadt, Germany) [47]. The contact angles were investigated using three measuring liquids: polar (distilled water), non-polar (diiodomethane), and intermediate (ethylene glycol). The SFE of the tested compositions was calculated based on Equation (1), where: *γS_p_* is the value of the polar component and *γS_d_* is the value of the dispersion component (mJ/m^2^):

(1) $$\gamma S=\gamma S_{p}+\gamma S_{d}$$

The degree of cross-linking was measured in accordance with the PN ISO 1817:2011 standard [48]. Samples weighing 30–40 mg were cut from the material then placed in water at room temperature for 48 h. Once the samples were swollen, they were dried and weighed. Analysis was carried out for five samples. The sample weights were used to calculate the values for equilibrium swelling Q_w_ (Equation (2)) and the degree of cross-linking α_c_ (Equation (3)):

(2) $$Q_{w}=\frac{\left(m_{\mathrm{sp}}-m_{s}\right)}{m_{s}}$$

(3) $$\alpha_{c}=\frac{1}{Q_{w}}$$

m_sp_—mass of the swollen sample [mg]; m_s_—mass of the dried sample after swelling [mg]; Q_w_—equilibrium swelling; α_c_—degree of cross-linking.

#### 3.3.6. Thermo-Oxidative Aging

To evaluate the effect of hermos-oxidative aging, measurements were made in accordance with ISO 2578: 1993 [47]. Paddle-shaped samples were placed in a hermos-forced thermal chamber for 7 days at 70 °C.

#### 3.3.7. UV-VIS Spectroscopy and Color Stability

After aging the samples were examined for color changes using a CIE Lab UV-Vis spectrophotometer. The CIE-LAB color space expresses the following characteristics of color: brightness, saturation, and hue. The test provides the color as described in the CIE-Lab space and in a system of three coordinates: L, a, and b, where L is the brightness parameter (maximum value of 100, representing a perfectly reflecting diffuser, minimum value of zero, representing the color black), a is the axis of spectra of red to green, and b is the axis of spectra of yellow to blue. The a and b axes have no specific numerical limitations. The change of color, dE∗ab, was calculated according to following Equation (4):

(4) $$\mathrm{dE}*\mathrm{ab}=\sqrt{\left(\Delta a^{2}\right)+\left(\Delta b^{2}\right)+\left(\Delta L^{2}\right)}$$

The color difference ∆E is determined according to PN-EN 105-J01:2002 and PN-EN-105-J03:2009 [49].The advantage of the CIELAB system is the ease with which colors can be compared. This is important for the production of products with complex color patterns.

#### 3.3.8. Soil Biodegradation

Biodegradable aging in soil was carried out in a climatic chamber using an HPP 108 (Memmert GmbH, Schwabach, Germany) in accordance with the PN-EN-ISO 846 standard. The samples were placed in universal soil for 14 days at 30 °C and 80% air humidity [50].

During microbiological decomposition, the change in mass of the samples was obtained. To determine the percentage of mass’ change the samples were weighed after obtainment, followed by weighing every 24 h until full material decomposition occurred. Then, the percentage of weight change was calculated using Equation (5). The experiment was conducted for three samples for every used blend in the experiment.

(5) $$\Delta m=\left|\frac{m_{0}-m_{a}}{m_{0}}\right|\cdot 100\%$$

Δm—percentage of mass’ change; m_0_—mass of the sample before aging; m_a_—mass of the sample after 24 h of aging.

## 4. Conclusions

Polysaccharide additives (starch and agarose) significantly improved the mechanical properties of the blend. Changing the alkaline environment had a positive effect on the biopolymer, with an increase in both tensile strength and relative tensile strength at break. Based on the results, the use of polysaccharidal additives in a gelatin matrix has great potential. The additives maintained the low thermal properties (softening point and glass transition temperature) and the same molecular structure of the basic biopolymer matrix, proved by FTiR analysis, while increasing its mechanical properties, surface energy, and the degree of cross-linking. In addition, SEM analysis showed little change in the topography of the samples compared to the basic material. The low softening and glass transition temperatures enable greener synthesis of the biopolymer compared to conventional plastics. Due to the nature of both the gelatin blend and additives themselves, the final material undergoes complete microbiological decomposition in the soil, as well as a significant change in properties due to thermo-oxidative aging. This confirms its environmental friendliness and degradability. Therefore, the product could potentially be used in packaging and single-use products.

## Figures and Tables

**Table 1:** Shore A hardness measurements with standard deviation.

Composition	H ± dH (^°^Sh)	T_s_ (MPa)	E_b_ (%)
**Control_0**	67.83 ± 8.84	5.83 ± 0.05	134.9 ± 8.5
**Control_0.1**	53.47 ± 1.77	4.09 ± 0.17	153.3 ± 5.7
**Control_0.5**	45.03 ± 1.85	3.54 ± 0.12	219.77 ± 10.4
**Control_1**	39.57 ± 1.19	2.01 ± 0.13	235.93 ± 8.3
**Agarose_0**	59.83 ± 3.04	1.27 ± 0.13	119.3 ± 9.9
**Agarose_0.1**	78.57 ± 2.35	3.51 ± 0.04	183.9 ± 14.9
**Agarose_0.5**	86.80 ± 5.03	3.64 ± 0.03	193.6 ± 7.5
**Agarose_1**	65.80 ± 1.41	2.01 ± 0.08	127.3 ± 13.4
**Starch_0**	20.67 ± 1.43	2.79 ± 0.03	120.1 ± 8.0
**Starch_0.1**	22.93 ± 2.31	3.57 ± 0.08	126.4 ± 5.9
**Starch_0.5**	32.43 ± 2.41	5.86 ± 0.11	159.4 ± 4.1
**Starch_1**	13.71 ± 1.01	1.17 ± 0.07	104.8 ± 5.1

**Table 2:** Comparison of polar and dispersion components and surface energies of samples synthesized with the addition of 0.5M sodium hydroxide.

Sample	Polar Component *γS_p_* (mN/m)	Dispersion Component *γS_d_* (mN/m)	Surface Energy S (mN/m)
**Control_0.5**	2.52 ± 0.26	33.85 ± 5.87	36.37 ± 6.01
**Agarose_0.5**	5.35 ± 1.15	40.02 ± 3.02	45.37 ± 4.15
**Starch_0.5**	2.10 ± 0.07	34.85 ± 5.01	36.86 ± 5.02

**Table 3:** Thermal properties of samples.

Sample	T_5%_ (°C)	T_peak 1 (DTG)_ (°C)	T_peak 2 (DTG)_ (°C)	∆C_p_ (J/g∙K)	T_g_ (°C)	T_s_ (°C)
**Control_0.5**	153 ± 2	257.11 ± 2	349.25 ± 2	0.251 ± 0.001	41.28 ± 2	34.17 ±1.75
**Agarose_0.5**	158 ± 2	273.76 ± 2	347.34 ± 2	0.547 ± 0.003	47.17 ± 2	39.51 ± 1.90
**Starch_0.5**	151 ± 2	258.90 ± 2	338.34 ± 2	0.302 ± 0.002	44.11 ± 2	39.43 ± 1.14

Legend: T_5%_—decomposition temperature at mass loss of 5%; T_p(DTG)_—max temperature of the maximum conversion factor on the DTG curve; ∆C_p_—heat capacity J/g∙K; T_g_—glass transition temperature. Standard deviation: T_5_, T_p (DTG)_ ± 2 °C; Δm _total_ ± 0.6%; Tg ± 2 °C.

**Table 4:** Results of equilibrium swelling analysis (Q_w_) and degree of cross-linkage (α_c_).

Blend Name	Q_w_ (-)	α_c_ (-)
**Control_0.5**	20.70 ± 0.26	0.050 ± 0.001
**Agarose_0.5**	7.52 ± 0.10	0.130 ± 0.002
**Starch_0.5**	14.08 ± 0.14	0.070 ± 0.001

**Table 5:** Analysis of color changes after thermo-oxidative aging.

Sample Name	dE∗ab	Before Aging	After Aging
**Control_0.5**	12.38	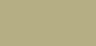	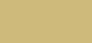
**Agarose_0.5**	17.67	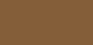	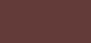
**Starch_0.5**	11.84	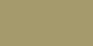	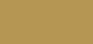

**Table 6:** Results of mechanical analysis (Shore hardness H; tensile strength T_S_; relative elongation at break E_b_) of biopolymer blends after thermo-oxidative aging.

Blend Name	H ± dH (°Sh)	T_s_ (MPa)	E_b_ (%)
**Control_0.5**	44.60 ± 3.30	5.09 ± 0.19	30.7 ± 4.5
**Agarose_0.5**	56.33 ± 2.84	5.99 ± 0.20	12.5 ± 2.7
**Starch_0.5**	56.80 ± 0.92	2.37 ± 0.09	10.6 ± 0.5

**Table 7:** Components used for blends’ synthesis. Amounts are presented in parts by weight (PBW).

Blend Name	Ingredients/Mass					
	Gelatin (g)	Glycerin (g)	Water (g)	NaOH 0.1M (g)	NaOH 0.5M (g)	NaOH 1M (g)
**Control_0**	60	40	70	-	-	-
**Control_0.1**				20	-	-
**Control_0.5**				-	20	-
**Control_1**				-	-	20

**Table 8:** Components used in compositions. Amounts are presented in parts by weight.

Blend Name	Ingredients/Mass							
	Gelatin (g)	Glycerin (g)	Water (g)	Agarose (g)	Starch (g)	NaOH 0.1M (g)	NaOH 0.5M (g)	NaOH 1M (g)
**Agarose_0**	60	40	70	5	0	*-*	-	-
**Agarose_0.1**						20	-	-
**Agarose_0.5**						*-*	20	-
**Agarose_1**						*-*	-	20
**Starch_0**				0	5	*-*	-	-
**Starch_0.1**						20	-	-
**Starch_0.5**						*-*	20	-
**Starch_1**						*-*	-	20
